# Altered Intra- and Inter-Network Functional Connectivity in Patients With Crohn’s Disease: An Independent Component Analysis-Based Resting-State Functional Magnetic Resonance Imaging Study

**DOI:** 10.3389/fnins.2022.855470

**Published:** 2022-03-02

**Authors:** Lu Li, Jie Ma, Xuyun Hua, Yan Zhou, Yage Qiu, Zhen Zhu, Yanling Zheng, Qian Xie, Zonghui Liang, Jianguang Xu

**Affiliations:** ^1^Department of Radiology, Jing’an District Centre Hospital of Shanghai, Fudan University, Shanghai, China; ^2^School of Rehabilitation Science, Shanghai University of Traditional Chinese Medicine, Shanghai, China; ^3^Department of Traumatology and Orthopedics, Yueyang Hospital, Shanghai University of Traditional Chinese Medicine, Shanghai, China; ^4^Department of Radiology, Renji Hospital, School of Medicine, Shanghai Jiao Tong University, Shanghai, China; ^5^Department of Radiology, Putuo People’s Hospital, Tongji University, Shanghai, China

**Keywords:** functional connectivity, independent component analysis, resting-state network, Crohn’s disease, brain-gut axis

## Abstract

**Background:**

Many studies have reported changes in the structure and function of several brain areas in patients with Crohn’s disease (CD). However, little is known about whether the possible functional connectivity of resting-state networks (RSNs) is altered in CD patients.

**Purpose:**

Aim to investigate the intra- and inter-network alterations between related RSNs in patients with CD and the potential relationships between altered neuroimaging and CD clinical indices.

**Materials and Methods:**

In this study, 20 CD patients and 22 age- and sex-matched healthy controls were included. All participants underwent functional magnetic resonance imaging examination. We used independent component analysis (ICA) to explore the changes in RSNs and evaluated functional connectivity between different RSNs using functional network connectivity (FNC) analysis, and Pearson correlation analysis was performed between altered intra- and inter-network functional connectivity and CD clinical index.

**Results:**

Six CD-related RSNs were identified *via* ICA, namely the high visual, prime visual, language, dorsal default mode, posterior insula, and precuneus networks. Compared to healthy controls, patients with CD showed significant changes in prime visual and language networks. Additionally, the functional connectivity (FC) values of the left calcarine within the prime visual network were negatively correlated with CD duration. The inter-alterations showed that a significantly increased FNC existed between the language and dorsal default mode networks.

**Conclusion:**

The results showed CD-related changes in brain functional networks. This evidence provides more insights into the pathophysiological mechanisms of brain plasticity in CD.

## Introduction

Crohn’s disease (CD) is a chronic inflammatory bowel disease (IBD) with an unclear pathogenesis that can affect the entire digestive tract. In recent years, with the urbanization of Asian countries, population mobility, and changes in dietary structure, the incidence in China has increased annually (Torres et al., 2017; Ng et al., 2018). As the disease progresses, multiple complications such as intestinal stenosis, perforation, and fistula may occur (Jostins et al., 2012; Uhlig, 2013; Loddo and Romano, 2015). Diseases can also invade various systems throughout the body and are often associated with extra-intestinal manifestations (EIM), such as arthritis, oral ulcers, and erythema nodosa. There is evidence that white matter lesions and neurological deficits in IBD patients may be an additional extraintestinal manifestation of the disease (Geissler et al., 1995). Patients with IBD often experience mood disorders, overreaction to stress, and imbalance of intestinal flora (Bonaz and Bernstein, 2013) and often endure mental symptoms such as anxiety and depression. Brain-gut interaction refers to the physiological or pathophysiological phenomenon in which the central nervous system and the intestine interact and control each other (Gracie et al., 2018), not only maintaining gastrointestinal homeostasis, but also affecting higher cognitive functions through neural pathways, cellular and humoral mediators (De Palma et al., 2014; Carabotti et al., 2015). Meanwhile, enteric microbiota may have an impact on nervous system *via* neural signaling, endocrine and immune mechanisms (Iweala and Nagler, 2006). In addition, microbial signaling molecules could interact directly with afferent nerve terminals during inflammation or stress (Furness and Clerc, 2000). Dysfunction of the brain-gut axis is considered to be the major causative factor in the development of CD (Mogilevski et al., 2019).

In this view, previous studies reported significant brain changes using structural and functional magnetic resonance imaging (fMRI) technology. Significant changes in gray matter (GM) structures in multiple brain regions were found in the study by Bao et al. (2015), and altered GM structures were associated with CD duration in specific regions. Nair et al. (2016) found that patients with CD had significant cortical thickening in the left superior frontal area. A study by Agostini et al. (2013a), CD patients demonstrated that the gray matter volume of the frontal lobe and cingulate cortex was reduced. However, Kornelsen et al. (2020) found no structural difference using voxel-based morphometry analysis, which showed increased FC between the frontal and parietal network and the salience network (SN), and the ICA results showed changes in the cerebellum, vision, and SN components. In addition, the fMRI study of CD applies an experimental research design to examine the processing of stress tasks or visceral stimulation or to analyze resting-state data to examine internal brain functions. Compared to controls, the stress task elicited greater neural activity in the midcingulate cortex and altered habituation to stress in CD patients (Agostini et al., 2013b,^2017^). Under uncertain, uncomfortable visceral sensations, CD patients show excessive reactivity in brain regions known to be involved in sensory, cognitive, and emotional aspects of pain processing (Rubio et al., 2016). At the same time, the resting-state MRI study also found some abnormal changes in the brain area. Fan et al. (2020) reported that the intrinsic functional connectivity of the amygdala decreases, and abnormal FC is associated with the duration of disease.

However, previous research has focused mainly on the local changes in blood oxygen level-dependent signals or structures. Abnormalities in information communication and interaction between brain areas in CD patients and the relationship between the alterations and the progression of the condition require in-depth study. To date, functional connectivity patterns within and between networks have be examined in few CD studies, which has been used more deeply in cognitive impairment and other brain impairment (Li et al., 2020, 2021). In this study, spatial independent component analysis (ICA) was used to identify brain networks (Beckmann et al., 2005). We aimed to study the functional connectivity change patterns across different brain networks.

## Materials and Methods

### Participants

This study was approved by the Ethics Committee of the Shanghai Jing’an Centre Hospital. All participants provided written informed consent. This was a prospective trial. Twenty patients with CD were enrolled between January and August 2020. All patients were evaluated by an experienced gastroenterologist, and medical records were reviewed to obtain endoscopic and hematological data and additional information. The inclusion criteria were as follows: 18–55 years of age, right-handed, and >6 years of education. The exclusion criteria were intestine-related abdominal surgery, use of psychotropic medications in the previous 6 months, pain syndromes, organic brain lesions, pregnancy, claustrophobia, or metallic implants.

Matched with age, gender, handedness, and education level, 22 healthy controls were recruited through advertisements. The adopted inclusion and exclusion criteria in the control group were the same as those in the CD group.

### Crohn’s Disease Clinical Measurement

The CDAI (Crohn’s disease activity index) (Best et al., 1979) was used to access the clinical condition of CD patients. The SDS (Self-rating depression scale) and SAS (Self-rating anxiety scale) were used to evaluate psychological level. Each scale comprises 20 items designed to assess depression symptoms and anxiety levels. In addition, the disease duration was recorded in months.

### Magnetic Resonance Imaging Data Acquisition

All fMRI data were acquired using a 3.0T MR scanner (SIEMENS MAGNETOM Prisma) with a 64-channel phase-array head coil. During scanning, participants were asked to stay awake with their eyes closed and ears plugged and avoid thinking of anything in particular. The 3D T1-weighted anatomical images were acquired in the sagittal orientation with the following parameters: TR = 1,800 ms, TE = 2.28 ms, slice thickness = 1 mm, flip angle = 8°, field of view = 256 × 256 mm^2^, matrix = 256 × 256, and number of slices = 160. Functional data were acquired using echo planar imaging sequence with the following parameters (multi-band, acceleration factor = 2): TR = 2,000 ms, TE = 30 ms, slice thickness = 2 mm, flip angle = 90°, field of view = 230 × 230 mm^2^, matrix = 64 × 64, number of slices = 70, and total volume = 220 was acquired in 8 min.

### Image Data Preprocessing

Based on Matlab 2018a operating platform and SPM 12.0 (Statistical Parametric Mapping),^1^ the toolbox for Data Processing and Analysis of Brain Imaging (DPABI)^2^ (Yan et al., 2016) was used to preprocess the rs-fMRI data. Preprocessing procedures included the following: (1) data at the first ten time points were removed to reach equilibrium and allow participants to adapt to the scanning environment, and the remaining 210 time points were used for preprocessing; (2) slice-time and head-motion were corrected, functional images were realigned to the first volume by Friston 24 motion correction procedure, and data with translation greater than 3.0 mm and rotation angle greater than 3° in all directions were eliminated; (3) normalization: the DARTEL approach (Goto et al., 2013) was used to register the corrected image, and normalized data were resampled to 3 mm × 3 mm × 3 mm isotropic voxels; and (4) spatial smoothing: a Gaussian smoothing kernel function with a full width at half maximum of 6 mm × 6 mm × 6 mm was applied to a spatially smooth fMRI image.

### Group Independent Component Analysis Analysis and Resting-State Networks Identification

Spatial ICA was conducted using the Group ICA functional MRI Toolbox (GIFT, version 4.0b).^3^ First, a two-level principal component analysis was employed for dimensionality reduction of the fMRI data, and 45 independent component (IC) maps were identified using the minimum description length criteria. Second, the data were decomposed using the Infomax algorithm. To increase the stability of ICs, we adopted the ICASSO algorithm 100 times (Himberg et al., 2004). Then, the group ICs (both spatial maps and time courses) were back-reconstructed using GICA for each subject (Calhoun et al., 2001). We determine whether a component is a meaningful RSN using the following steps. We eliminated those components that were mainly distributed in white matter, ventricles, or susceptibility artifacts through visual observation. Spectrum analysis was also performed on the time course corresponding to each independent component. Because the energy of the resting state network is generally concentrated below 0.1 Hz, we eliminated those components with most of the energy distributed above 0.1. Next, the method of similarity analysis with template spatial matching was used to determine the brain network. The 14 reference networks were described in a previous study (Shirer et al., 2012).

For each selected RSN, the group spatial map was determined for all subjects using a one-sample *t*-test (*P* < 0.05, FWE corrected), and the significant clusters of one-sample *t*-test of IC results were defined as a network mask. The functional connectivity changes in each RSN between groups were investigated using a two-sample *t*-test (*P* < 0.01 at cluster level, AlphaSim corrected) and the mask was used to avoid false-positive results.

### Functional Network Connectivity Analysis Between Resting-State Networks

For internetwork functional connectivity analysis, the temporal correlations among all RSNs were calculated using the constrained maximal lag correlation approach. FNC correlation maps from the two groups were generated from all possible RSN combinations. A two-sample *t*-test (*P* < 0.05, FDR corrected) for group comparisons was performed using pair-wise combinations.

### Statistical and Correlation Analysis

The clinical characteristics were analyzed using SPSS (version 20.0; SPSS Inc., Chicago, IL, United States). Independent *t*-tests were performed for age, and the chi-square test was used to compare gender differences. A Pearson correlation analysis was applied between the intra- and inter-network FC of significant group differences and clinical measures across all CD patients. All statistical significance thresholds were set at *P* < 0.05.

## Results

### Clinical Characteristics

The clinical and demographic characteristics of all participants are shown in Table 1, and there was no difference in age and sex between the two groups (*P* > 0.05).

**TABLE 1:** Clinical characteristics of participants at baseline in each group.

Characteristic	CD patients (*n* = 20)	Healthy controls (*n* = 22)	*P*-value
Gender (male/female)	8/12	10/12	0.721^a^
Age (years)	33.00 ± 13.35	37.82 ± 7.40	0.151^b^
CDAI	196.03 ± 33.43	N/A	
SAS	35.45 ± 7.32	N/A	
SDS	41.60 ± 7.38	N/A	
Disease duration (months)	61.00 ± 70.58	N/A	

*^a^χ^2^ test; ^b^Independent t-test for continuous data (mean ± SD). CD, Crohn’s disease; CDAI, CD activity index; SAS, Self-rating Anxiety Scale; SDS, Self-rating Depression Scale; and N/A, not applicable.*

### Identification of Resting-State Networks in Patients With Crohn’s Disease and Healthy Controls

**FIGURE 1:**
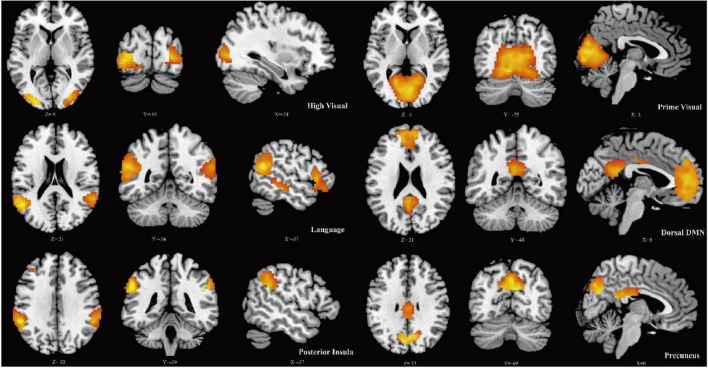
Spatial maps for six resting-state networks (RSNs). Each RSN map was obtained using a one-sample *t*-test across all individual IC patterns.

Six ICs were determined using the ICA algorithm, which were classified into the following six large-scale networks: high visual network, prime visual network, language network, dorsal default mode network, posterior insula network, and precuneus network. Further analysis was performed based on these RSNs (Figure 1).

### Functional Connectivity Analysis Within Resting-State Networks

**FIGURE 2:**
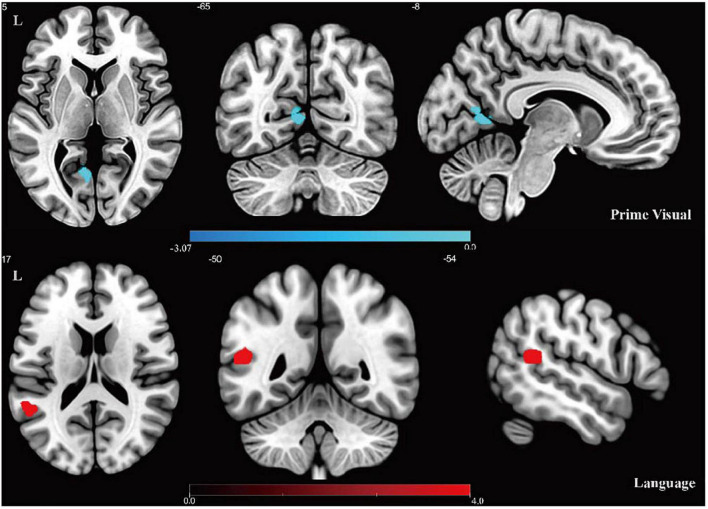
Results of intra-network FC analysis. Altered FC in the prime visual network and language network: the cool color denotes lower functional connectivity in the CD group compared with the control group, and the hot color denotes higher functional connectivity in the CD group. Color bar presents *t*-value.

The results of two-sample *t*-tests between the intra-networks of the CD and control groups are shown in Figure 2 and Table 2. Compared with healthy controls, the prime visual network showed decreased functional connectivity in the left calcarine (CAL.L), while the language network showed increased functional connectivity in the left middle temporal gyrus (two-tailed, cluster level *P* < 0.01, AlphaSim corrected).

**TABLE 2:** Differences in intra-network FC between CD group and controls.

RSNs	Region label	Voxel size	*t*-value	Peak MNI coordinates		
				*x*	*y*	*z*
**Prime visual**						
Negative	Left calcarine	28	−3.072	−9	−66	12
**Language**						
Positive	Left middle temporal	36	3.923	−51	−51	15

*Negative, CD < healthy control; Positive, CD > healthy control; MNI, Montreal Neurological Institute.*

### Functional Network Connectivity Analysis Between Groups

**FIGURE 3:**
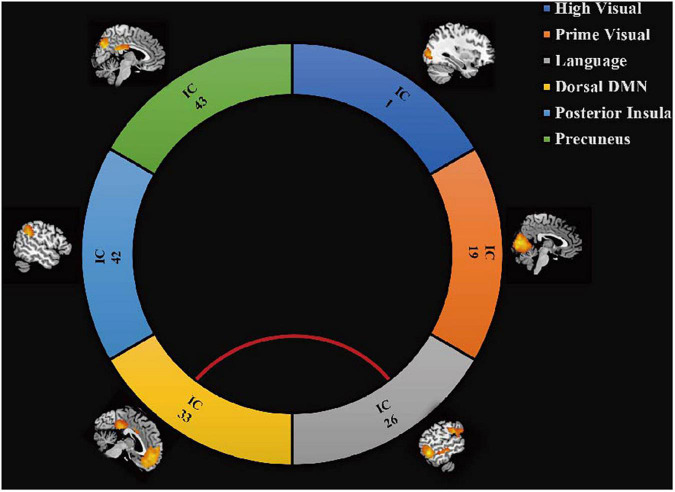
Differences in brain functional network connectivity between RSNs. Increased connectivity strength in the CD group is displayed between language network and dDMN (red line, *t* = 2.78, *P* = 0.008, FDR corrected).

The results of the functional network connectivity (FNC) analyses for different RSNs are shown in Figure 3. Compared with healthy controls, significantly increased connectivity was found between the language network and dorsal default mode network (DMN) (*P* < 0.05, FDR corrected).

### Correlation Analysis

**FIGURE 4:**
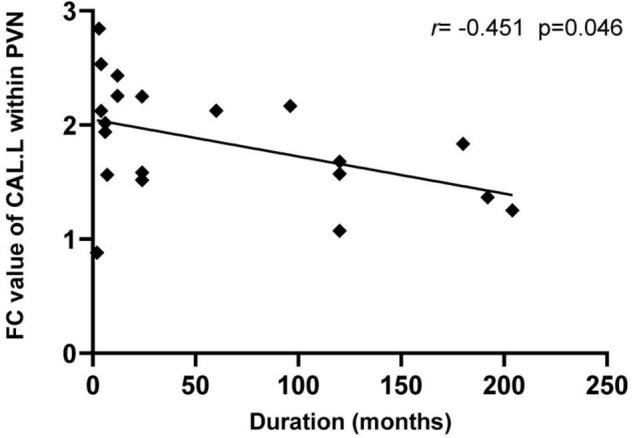
Relationship between the neuroimaging findings and CD clinical characteristics. The intra-network FC values of the left CAL showed a negative correlation with the duration of CD patients (*r* = –0.451, *p* = 0.046). CAL, calcarine; CD, Crohn’s disease; and PVN, prime visual network.

In the CD group, the CAL.L-related connectivity strength within the prime network was significantly negatively correlated with disease duration (*r* = −0.451, *P* = 0.046, Figure 4). However, there were no other significant correlations between CD clinical measurements (CDAI, SAS, and SDS) and alterations in neuroimaging (*P* > 0.05).

## Discussion

This study is based on resting-state fMRI (rs-fMRI) data, aiming to study the interaction changes of the intra- and inter-brain networks in CD patients using ICA and FNC algorithms. Our research suggests that the connectivity within RSNs in the CD group and functional connections between RSNs changed. These alterations indicate functional impairment and remodeling within and between RSNs in patients with CD. Previous studies have focused mostly on changes in plasticity of focal brain regions. However, it is meaningful to study the interaction mode of RSNs as the brain runs as a whole with complex internal networks.

There are complex anatomical structures corresponding to specific functions in each RSN. In our study, ICA results showed that there were differences between the two RSNs in the CD group, suggesting changes in visual and language processes. The results showed that CD patients had decreased FC of the prime visual network with CAL.L but increased FC of the language network with the left middle temporal gyrus.

Although there is no direct evidence or research showing that visual network changes are involved in the CD process, some studies have found that CD patients have abnormalities in vision-related brain areas, including differences in the thickness of the occipital cortex (Nair et al., 2016) and structural differences in the left lingual gyrus (Thomann et al., 2016), changes in the vision components (Kornelsen et al., 2020). Patients with IBD show significantly increased functional connectivity within the medial visual network bilaterally in the visual cortex after transcranial direct current stimulation treatment (Neeb et al., 2019). Similar changes have been reported in other chronic pain-related diseases, such as persistent somatic pain disorder (Zhao et al., 2017), knee osteoarthritis (Pujol et al., 2017), and migraine (Liu et al., 2012), we speculate that long-term stimulation of chronic pain will cause visual-related changes in the brain. It has also been found that patients with chronic low back pain have significantly enhanced connectivity between the primary visual network and the somatosensory/motor areas, and this result was confirmed by the machine learning method (Shen et al., 2019). In addition, our correlation analysis also found that the functional connectivity strength of the CAL.L area within the visual network was negatively correlated with disease duration. Patients with CD are stimulated by chronic abdominal pain for a long time, which may be the cause of abnormal performance in their visual areas. It is difficult to fully guarantee the eyes opened state of the participants during the scan, which may be bias in the results of the study.

Language processing relies on the coordinated activities of multiple brain regions, especially in the frontal and temporal lobes of the left hemisphere (Wagner et al., 2014). A study has found that CD patients in remission also show a reduction in age-related verbal fluency (VF) task asymmetry. After a VF task, the activation patterns of young CD patients and the young control group participants were significantly different; however, they were similar to those of the old healthy control group participants, which might indicate if CD patients show accelerated age-like effects (Nair et al., 2019). Under normal circumstances, the VF task in young people shows the activation pattern in the left hemisphere, while elderly people have a bilateral activation pattern in the same task (Cabeza et al., 2002; Meinzer et al., 2008, 2009), which indicates a compensation or dedifferentiation mechanism. These results suggest that IBD patients experience accelerated neuro-aging changes in the language network. A special study compared asymptomatic adolescent IBD patients with a control group and found that those with IBD had lower average grade points and worse performance in some subjects in school (Mackner et al., 2012). Dancey et al. (2009) showed that compared to healthy controls and controls with IBS, adult patients with IBD demonstrated lower verbal IQ scores, indicating that the disease process of IBD could be closely related to these effects.

In other studies, CD patients have been shown to have a significant association between cognitive performance (verbal, executive, and others) and disease activity (Golan et al., 2016). There is evidence that the increase in pro-inflammatory cytokines in patients with CD has a suggestive effect on cognitive decline; however, this does not prove causality (Dziedzic, 2006). It is worth noting that our FNC research results show that the connectivity between the language network and the dorsal DMN is enhanced. The results indicate that in CD patients, the language network and dorsal DMN cooperate more closely. Chronic visceral pain in patients with functional gastrointestinal disorders and IBD is closely related to the DMN, and chronic pain has been reported to cause functional reorganization of the default mode network (Farmer et al., 2012; Qi et al., 2016; Kano et al., 2018). This change may be due to the early functional compensation of the patient’s language network damage and the increased functional connectivity with the dorsal DMN, which may reflect an adaptive or self-regulating mechanism.

## Limitations

Because of the complexity of CD and the limited sample size, we failed to explore possible differences in patient group with different disease features. Larger sample size is necessary to obtain more convincing results. It is worth noting that some of the previous findings are more pronounced in patients with EIM, and future research needs to focus on more disease-related factors of CD, such as disease burden, activity or severity, and drug use, to determine their different effects on brain activation patterns. The cognitive assessment, the fecal samples collection and gut microbiota analysis for further investigation is also necessary.

## Conclusion

In conclusion, our study demonstrated brain alterations in patients with CD in the visual and language networks. Moreover, FC alteration in the CAL.L may reflect the degree of sustained impairment of the disease duration. In addition, the enhanced FNC suggests that there may be abnormal activation or compensation in early stage. The findings of this study can provide additional evidence to further understand the role of the brain-gut interaction in CD, but the specific mechanism needs to be further studied.

## Data Availability Statement

The original contributions presented in the study are included in the article/supplementary material, further inquiries can be directed to the corresponding authors.

## Ethics Statement

The studies involving human participants were reviewed and approved by the Ethics Committee of the Shanghai Jing’an Centre Hospital. The patients/participants provided their written informed consent to participate in this study.

## Author Contributions

ZL and YZ designed the study. YQ, ZZ, YLZ, and QX contributed to the data collection. LL, JM, and XH visualized the data, carried out the formal analysis, and wrote the manuscript. ZL and JX approved the final manuscript. All authors contributed to the article and approved the submitted version.

## Conflict of Interest

The authors declare that the research was conducted in the absence of any commercial or financial relationships that could be construed as a potential conflict of interest.

## Publisher’s Note

All claims expressed in this article are solely those of the authors and do not necessarily represent those of their affiliated organizations, or those of the publisher, the editors and the reviewers. Any product that may be evaluated in this article, or claim that may be made by its manufacturer, is not guaranteed or endorsed by the publisher.

## Footnotes

1
http://www.fil.ion.ucl.ac.uk/spm

2
http://www.rfmri.org/dpabi

3
http://mialab.mrn.org/software/gift
